# The Ameliorative Effect of Coumarin on Copper Toxicity in *Citrus sinensis*: Insights from Growth, Nutrient Uptake, Oxidative Damage, and Photosynthetic Performance

**DOI:** 10.3390/plants13243584

**Published:** 2024-12-22

**Authors:** Wei-Lin Huang, Hui Yang, Xu-Feng Chen, Fei Lu, Rong-Rong Xie, Lin-Tong Yang, Xin Ye, Zeng-Rong Huang, Li-Song Chen

**Affiliations:** College of Resources and Environment, Fujian Agriculture and Forestry University, Fuzhou 350002, China; 2210807003@fafu.edu.cn (W.-L.H.); 52308031045@fafu.edu.cn (H.Y.); 2210807010@fafu.edu.cn (X.-F.C.); 22308007010@fafu.edu.cn (F.L.); rongrxie@fafu.edu.cn (R.-R.X.); talstoy@fafu.edu.cn (L.-T.Y.); yexin1000@fafu.edu.cn (X.Y.); huangzengrong@fafu.edu.cn (Z.-R.H.)

**Keywords:** CO_2_ assimilation, chlorophyll *a* fluorescence (OJIP) transient, nutrient balance, reactive oxygen species

## Abstract

Excessive copper (Cu) has become a common physiological disorder restricting the sustainable production of citrus. Coumarin (COU) is a hydroxycinnamic acid that can protect plants from heavy metal toxicity. No data to date are available on the ameliorative effect of COU on plant Cu toxicity. ‘Xuegan’ (*Citrus sinensis* (L.) Osbeck) seedlings were treated for 24 weeks with nutrient solution containing two Cu levels (0.5 (Cu0.5) and 400 (Cu400) μM CuCl_2_) × four COU levels (0 (COU0), 10 (COU10), 50 (COU50), and 100 (COU100) μM COU). There were eight treatments in total. COU supply alleviated Cu400-induced increase in Cu absorption and oxidative injury in roots and leaves, decrease in growth, nutrient uptake, and leaf pigment concentrations and CO_2_ assimilation (A_CO2_), and photo-inhibitory impairment to the whole photosynthetic electron transport chain (PETC) in leaves, as revealed by chlorophyll *a* fluorescence (OJIP) transient. Further analysis suggested that the COU-mediated improvement of nutrient status (decreased competition of Cu^2+^ with Mg^2+^ and Fe^2+^, increased uptake of nutrients, and elevated ability to maintain nutrient balance) and mitigation of oxidative damage (decreased formation of reactive oxygen species and efficient detoxification system in leaves and roots) might lower the damage of Cu400 to roots and leaves (chloroplast ultrastructure and PETC), thereby improving the leaf pigment levels, A_CO2_, and growth of Cu400-treated seedlings.

## 1. Introduction

Copper (Cu) is not only a micronutrient required by plants, but also a heavy metal (HM). It works as a cofactor in enzymes including laccase, plastocyanin, cytochrome oxidase, ascorbate (ASC) oxidase, amino oxidase, superoxide dismutase (SOD), and polyphenol oxidase [1]. Therefore, Cu is involved in numerous processes, such as photosynthesis, respiration, redox reaction, and detoxification [2,3]. Like the other HMs, however, Cu will cause toxicity to most agricultural crops when its level in leaves exceeds 20–30 μg g^−1^ DW [2].

Anthropogenic activities have led to a significant input of HMs into agricultural soils, especially in permanent cultivation such as orchards [4]. Due to long-term foliar spraying of Cu-containing fungicides to prevent diseases and pests and/or soil application of Cu-containing fertilizer, excessive Cu has become a universal physiological disorder restricting the sustainable production of citrus in some old orchards, especially in acidic soil. Even worse, Cu toxicity in citrus is on the rise [5,6,7,8,9]. The application of Cu-containing fungicides can be traced back to the late 19th century [9]. About 30 years after their application started on citrus in Florida, USA, a widespread Cu toxicity developed [6]. In China, excessive Cu is one of the main soil nutrient problems in the main citrus-producing areas [10]. Li et al. [11] found that 70% and 28% of *Citrus grandis* orchards in Pinghe, Fujian, China, were excess in the foliar Cu and soil available Cu concentrations, respectively, and 90% of the orchard soils had a pH of less than 5.0. The symptoms of Cu toxicity on citrus trees include iron (Fe) deficiency chlorosis of young leaves, reduced growth, and poorly developed and darkened root systems with rotten and dead fibrous roots [12,13].

Copper is preferentially accumulated in the roots of Cu-exposed plants [2]. Higher concentrations of Cu can restrict root growth and damage root function. The damaged roots, in turn, lead to nutrient deficiency and imbalance, thereby impairing leaf photosynthetic performance and inhibiting plant growth [8,14,15,16]. Also, Cu toxicity can create an imbalance between the formation and removal of reactive oxygen species (ROS), resulting in over-accumulation of ROS in leaves and roots and causing oxidative damage to them [7,17,18].

The exogenous application of phenolic compounds (phenolic acids) can enhance plant tolerance against abiotic (HM) stress [19,20,21,22,23]. In addition to playing a role in ROS detoxification, phenolic compounds can also lower Cu toxicity in plant cells by forming stable, non-toxic chelates with Cu [24,25]. Exogenous application of phenolic compounds (gallic acid, phenolic acid (salicylic acid), and resveratrol) can alleviate Cu toxicity in plants by inhibiting Cu uptake and root-to-shoot Cu translocation, improving plant water status and reducing oxidative stress [21,26,27]. Coumarin (COU) is a hydroxycinnamic phenolic acid with significant antioxidant potential [28]. Most vascular plants produce COUs to protect them from pathogenic infections and other adverse conditions [29]. As is well known, COUs are related to the acquisition of Fe, and Fe starvation can stimulate their biosynthesis and secretion by roots [30]. The application of 50 μM COU to *Poncirus trifoliata* roots through solution culture alleviated high pH-induced Fe deficiency yellowing [31]. Excess of HMs (Cu, zinc (Zn), and manganese (Mn)) can cause Fe deficiency chlorosis in younger leaves [5,22,32]. Like Fe deficiency, excessive Zn in *Arabidopsis* seedling growth medium can also induce COU biosynthesis and release [32]. There is evidence showing that priming sorghum seeds with 100 mg L^−1^ COU and/or foliar application of 100 mg L^−1^ COU can alleviate sorghum salt stress by enhancing the accumulation of antioxidant compounds, activities of antioxidant enzymes, and photosynthesis, and their combined application has a better ameliorative effect than the individual one [23]; furthermore, pre-treating tomato seedling roots with 20 and 30 μM COU can confer tomato salt tolerance by reducing oxidative injury due to enhanced antioxidant system and reduced production of ROS and by maintaining ion (Na^+^, K^+^, Ca^2+^, and Mg^2+^) homeostasis and balance [33]. However, there are very limited data on the mitigation of HM toxicity by COU. In a study, Shad et al. [22] found that priming sesame seeds with 50, 100, and 150 mg L^−1^ COU for 24 h mitigated the inhibitory effect of excessive Mn on seedling growth by improving nutrient acquisition and photosynthesis and reducing Mn uptake and oxidative damage. The ameliorative effect of COU was more effective at 50 and 100 mg L^−1^ than at 150 mg L^−1^. However, no data to date are available on the ameliorative effect of COU on plant Cu toxicity.

Most commercial citrus trees are planted in humid and sub-humid regions, where soil acidification and high-soil-available Cu concentrations are common [10]. In a recent study from our laboratory, Ren et al. [34] identified eight upregulated and two downregulated COUs from roots of excessive Cu-treated *C. grandis* seedlings. These upregulated COUs might function in the adaptation of roots to Cu toxicity. The present study was undertaken to investigate the effects of Cu-COU interactions on growth, leaf photosynthetic performance, root, stem, and leaf nutrient levels, and H_2_O_2_ production rate (HPR), antioxidant enzyme activities, and malondialdehyde (MDA) concentrations in roots and leave of ‘Xuegan’ (*Citrus sinensis *(L.) Osbeck) seedlings. Our aim was to test the hypothesis that exogenous application of COU reduced the inhibitory action of excessive Cu on seedling growth through reducing Cu absorption and oxidative injury and improving plant nutrient status (homeostasis and balance) and photosynthetic performance.

## 2. Results

### 2.1. Effects of Cu-COU Interactions on Seedling Growth

**Figure 1:**
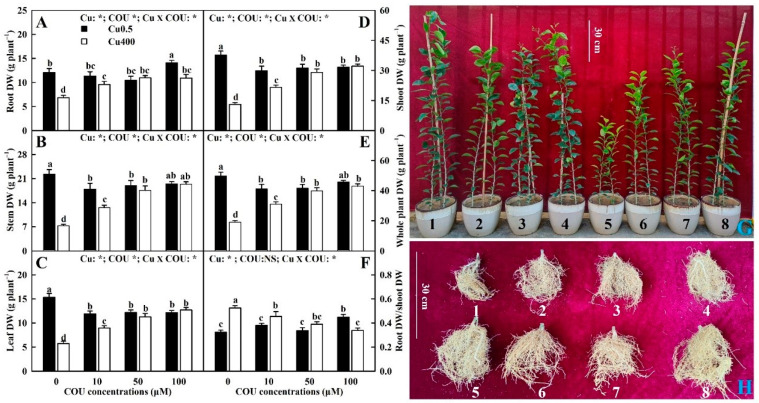
Effects of Cu-COU interactions on the mean (±SE, *n* = 10) root (**A**), stem (**B**), leaf (**C**), shoot (**D**), and whole plant (**E**) DW, root DW/shoot DW ratio (**F**), and shoot (**G**) and root (**H**) growth of *Citrus sinensis* seedlings. Significant differences were analyzed by two ANOVA and followed by the least significant difference (LSD) at *p* < 0.05. Error bars with different letters are significant different at *p* < 0.05. *, significant difference at *p* < 0.05; NS, non-significant difference. COU, coumarin; DW, dry weight; 1, 0.5 μM Cu + 0 μM COU; 2, 0.5 μM Cu + 10 μM COU; 3, 0.5 μM Cu + 50 μM COU; 4, 0.5 μM Cu + 100 μM COU; 5, 400 μM Cu + 0 μM COU; 6, 400 μM Cu + 10 μM COU; 7, 400 μM Cu + 50 μM COU; and 8, 400 μM Cu + 100 μM COU.

A shown in Figure 1A–F, 400 μM Cu (Cu400) significantly decreased the root, stem, leaf, shoot, and whole plant dry weight (DW) at 0 μM COU (COU0) and 10 μM COU (COU10), especially at COU0. The only exception that Cu400 did not significantly alter the root DW at COU10. On the contrary, Cu400 significantly increased the root DW/shoot DW (R/S) at COU0 and COU10, especially at COU0. However, Cu400 had no significant impacts on these six parameters at 50 μM COU (COU50) and 100 μM COU (COU100), except that it significantly decreased the root DW and the R/S at COU100. At Cu400, the whole plant, shoot, leaf, stem, and root DW increased as COU concentrations elevated from 0 to 50 μM, then kept stable with the further increment in COU supply, while the R/S decreased with the increase in COU supply. At 0.5 μM Cu (Cu0.5), the root DW and the R/S of seedlings treated with COU100 were significantly higher than those treated with COU0, COU10, or COU50, while the whole plant, shoot, leaf, and stem DW of seedlings treated with COU0 were significantly higher than those treated with COU10, COU50, or COU100, except that the whole plant and stem DW of seedlings treated with COU0 were similar to those treated with COU100. In addition to alleviating the decrease in seedling growth induced by Cu400, COU also reduced the yellowing of young leaves (Figure 1G) and the decay and death of fibrous roots (Figure 1H) caused by Cu400.

### 2.2. Effects of Cu-COU Interactions on Nutrient Status in Seedlings

**Figure 2:**
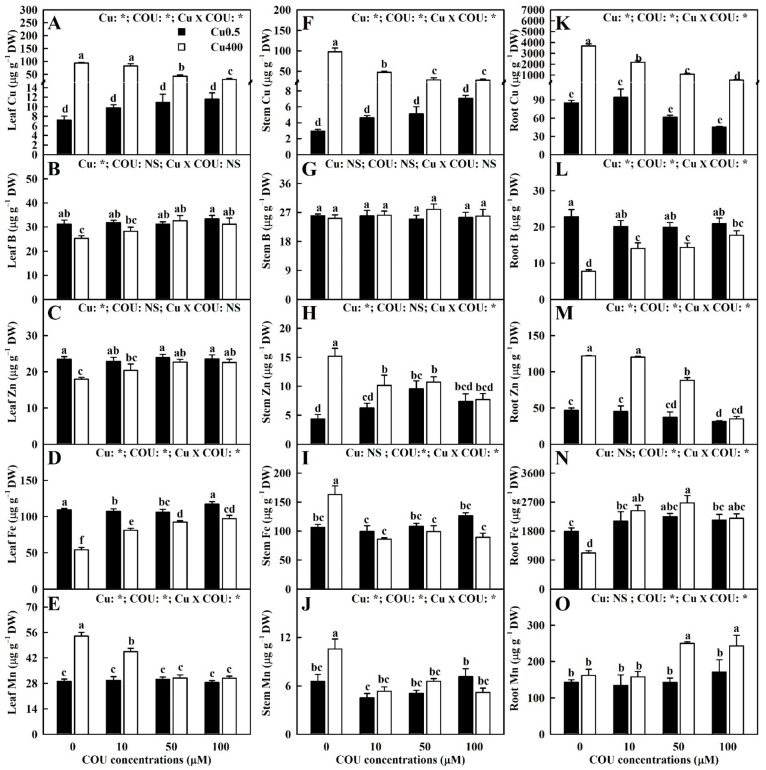
Effects of Cu-COU interactions on the mean (±SE, *n* = 4) concentrations of micronutrients in leaves (**A**–**E**), stems (**F**–**J**), and roots (**K**–**O**). Significant differences were analyzed by two ANOVA and followed by the LSD at *p* < 0.05. Error bars with different letters are significant different at *p* < 0.05. *, significant difference at *p* < 0.05; NS, non-significant difference.

It was found that Cu400 significantly elevated the leaf Cu level by 1200%, 742%, 295%, and 165%, the stem Cu level by 3221%, 930%, 471%, and 301%, and the root Cu level by 4228%, 2215%, 1666%, and 1109% at COU0, COU10, COU50, and COU100, respectively. The Cu levels in leaves, stems, and roots of Cu400-treated seedlings decreased with the increasing COU supply, but COU supply did not significantly change the Cu levels in leaves, stems, and roots of Cu0.5-treated seedlings (Figure 2A,F,K).

The results indicate that COU supply mitigated the Cu400-induced decreases in the boron (B), Zn, Fe concentrations in leaves and the B and Fe concentrations in roots, as well as the Cu400-induced increases in the Mn concentration in leaves, the Zn, Fe, and Mn concentrations in stems, and the Zn concentration in roots. Notably, Cu400 significantly increased the Mn concentration in roots at COU50 and COU100, but not at COU0 and COU10. At Cu400, COU supply significantly increased the B, Zn, and Fe concentrations in leaves and the B, Fe and Mn concentrations in roots, while it significantly decreased the Mn concentration in leaves, the Zn, Fe, and Mn concentrations in stems, and the Zn concentration in roots. At Cu0.5, COU addition did not significantly change their concentrations in leaves, stems, and roots, with a few exceptions. Cu-COU interactions had no significant effects on the B concentrations in stems (Figure 2B–E,G–J,L–O).

**Figure 3:**
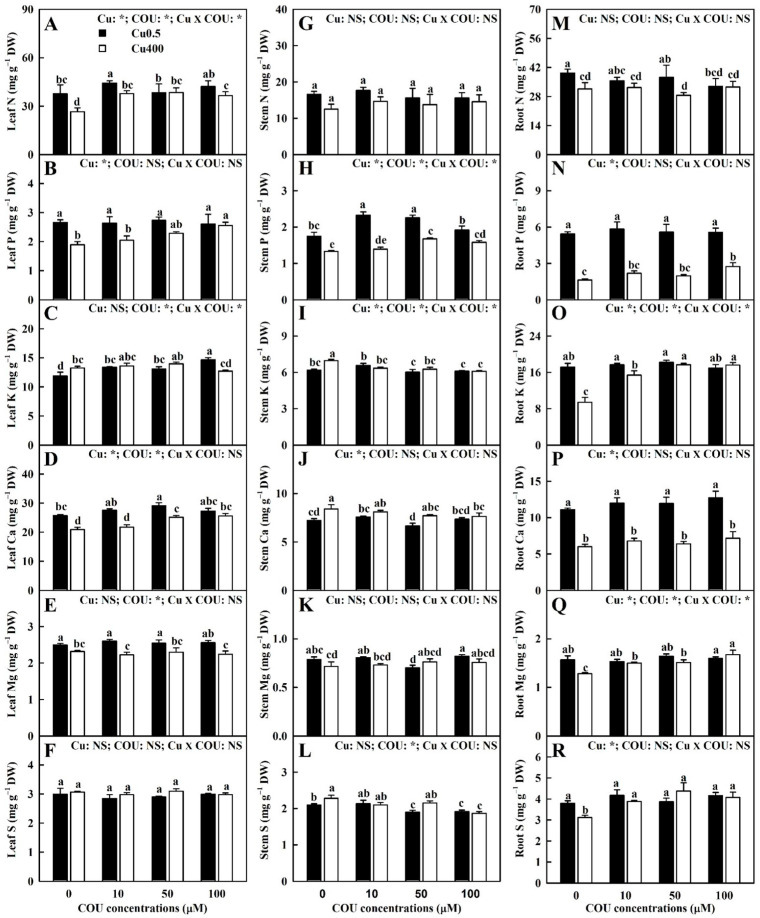
Effects of Cu-COU interactions on the mean (±SE, *n* = 4) concentrations of macronutrients in leaves (**A**–**F**), stems (**G**–**L**), and roots (**M**–**R**). Significant differences were analyzed by two ANOVA and followed by the LSD at *p* < 0.05. Error bars with different letters are significant different at *p* < 0.05. *, significant difference at *p* < 0.05; NS, non-significant difference.

It was found that COU supply mitigated the Cu400-induced decreases in the nitrogen (N), phosphorus (P), and calcium (Ca) levels in leaves, the P level in stems, and the P, K, Ca, magnesium (Mg), and sulfur (S) levels in roots, as well as the Cu400-induced increases in the potassium (K), Ca, and S levels in stems. Cu400 slightly reduced or did not significantly affect the Mg level in leaves and the N level in roots. Cu400 significantly increased the K level in leaves at COU0 and significantly decreased its level at COU100. Cu400 had no significant effects on the Mg level in stems. At Cu400, COU addition significantly increased the N, P, and Ca levels in leaves, the P level in stems, and the P, K, Ca, Mg, and S levels in roots, significantly decreased the K, Ca, and S levels in stems, and did not significantly alter the K and Mg levels in leaves, except for a slight decrease in the K level at COU100, the Mg level in stems, and the N level in roots. At Cu0.5, COU addition had no significant effects on the following: the N, P, Ca, and Mg levels in leaves, except for a slight increase in the N (Ca) level at COU10 (COU50); the K, Ca, and Mg levels in stems, except for a slight decrease (increase) in the Mg and Ca (K) levels at COU50 (COU10); and the concentrations of these six nutrients in roots, except for a slight decrease in the N level at COU100. At Cu0.5, COU addition slightly increased (decreased) the K (S) level in leaves (stems), and the P concentration in stems was significantly higher at COU10 and COU50 than at COU0 and COU100. Cu-COU interactions had no significant effects on the S (N) concentration in leaves (stems) (Figure 3).

**Figure 4:**
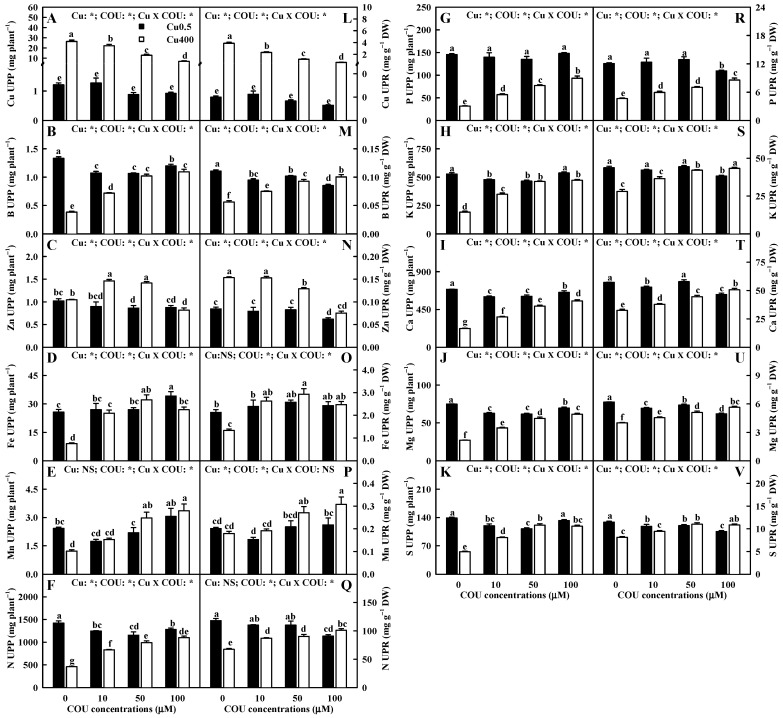
Effects of Cu-COU interactions on the mean (±SE, *n* = 4) nutrient UPP (**A**–**K**) and UPR (**L**–**V**). UPP, uptake per plant; UPR, uptake per root DW. Significant differences were analyzed by two ANOVA and followed by the LSD at *p* < 0.05. Error bars with different letters are significant different at *p* < 0.05. *, significant difference at *p* < 0.05; NS, non-significant difference.

It was observed that Cu400 significantly increased the Cu uptake per plant (UPP) by 2092%, 1652%, 1377%, and 654% and the Cu uptake per root DW (UPR) by 3779%, 1976%, 1308%, and 875% at COU0, COU10, COU50, and COU100, respectively. At Cu400, the Cu UPP and UPR decreased with the increase in COU supply, but at Cu0.5, the supply of COU did not significantly change these two parameters (Figure 4A,L).

The results indicate that Cu400 significantly decreased or did not significantly change the B and Fe UPP (UPR), except that Cu400 increased the B URP at COU100, but it significantly increased or did not significantly alter the Mn and Zn UPP (UPR), except that Cu400 significantly decreased the Mn UPP at COU0. At Cu0.5, the B UPP and the B and Zn UPR were significantly higher at COU0 than at COU100, but the Fe UPP was significantly higher at COU100 than at COU0. At Cu400, the B, Fe, and Mn UPP and UPR were significantly higher at COU100 than at COU0, but the Zn UPP and UPR were significantly higher at COU0 than at COU100 (Figure 4B–E,M–P).

As shown in Figure 4F–K,Q–V, the decrease in the Mg, Ca, N, P, K, and S UPP (UPR) induced by Cu400 decreased with an increase in COU supply, with a few exceptions. At Cu400, the Mg, Ca, N, P, K, and S UPP (UPR) increased with the increase in COU supply with a few exceptions, while at Cu0.5, the Mg, Ca, N, P, K, and S UPP (UPR) were significantly higher at COU0 than at COU100 or similar between the two.

**Figure 5:**
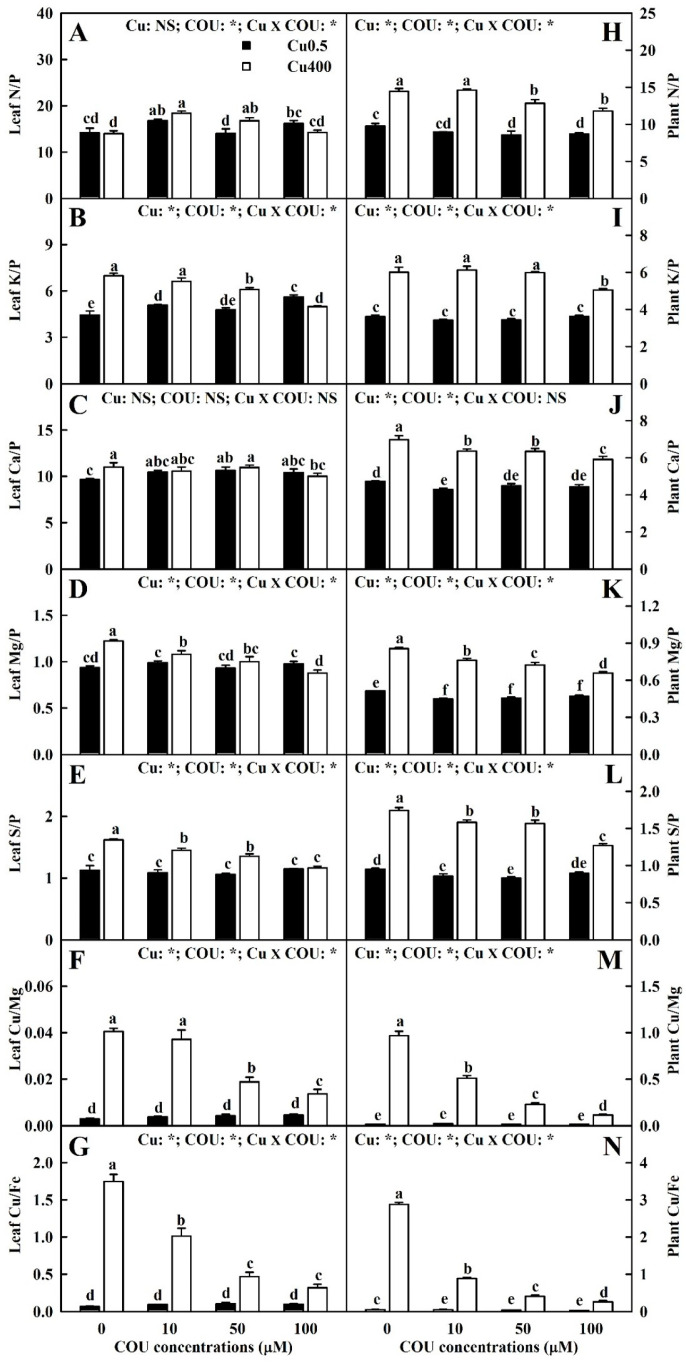
Effects of Cu-COU interactions on the mean (±SE, *n* = 4) ratios of N, K, Ca, Mg, and S (Mg and Fe) concentrations to P (Cu) concentration in leaves (**A**–**G**) and ratios of N, K, Ca, Mg, and S (Mg and Fe) UPP to P (Cu) UPP (**H**–**N**) in *C. sinensis* seedlings. Significant differences were analyzed by two ANOVA and followed by the LSD at *p* < 0.05. Error bars with different letters are significant different at *p* < 0.05. *, significant difference at *p* < 0.05; NS, non-significant difference.

Figure 5 displayed the effects of Cu-COU interactions on the ratios of leaf N, K, Ca, Mg, and S concentrations to leaf P concentration and the ratios of leaf Cu concentration to leaf Mg and Fe concentrations (hereinafter referred to as leaf N/P, K/P, Ca/P, Mg/P, S/P, Cu/Mg, and Cu/Fe), as well as the ratios of N, K, Ca, Mg, and S UPP to P UPP and the ratios of Cu UPP to Mg and Fe UPP (hereinafter referred to as plant N/P, K/P, Ca/P, Mg/P, S/P, Mg/Cu, and Fe/Cu). COU addition reduced Cu400-induced increase in the leaf K/P, Mg/P, and S/P; Cu400 did not significantly affect the leaf N/P and Mg/P, except that Cu400 significantly increased the leaf N/P at COU50 and leaf Ca/P at COU0. At Cu0.5, there was no significant difference in the leaf N/P, Ca/P, Mg/P, and S/P between COU0 and COU100 treatments, but the leaf K/P was significantly higher at COU100 than at COU0 (Figure 5A–E). COU supply reduced Cu400-induced increase in the plant N/P, K/P, Ca/P, Mg/P, and S/P. At Cu0.5, COU supply did not significantly alter these five parameters except for a slight increase in the plant N/P, Ca/P, Mg/P, and S/P at COU0, but at Cu400, these five parameters decreased with the increase in COU supply (Figure 5H–L).

As shown in Figure 5F,G,M,N, COU supply reduced Cu400-induced increase in the leaf Cu/Mg and leaf Cu/Fe, as well as the plant Cu/Mg and Cu/Fe. At Cu0.5, the supply of COU had no significant impacts on the leaf Cu/Mg and Cu/Fe, as well as the plant Cu/Mg and Cu/Fe. At Cu400, these four parameters declined with the rise in COU supply.

Figure S1 shows the effects of Cu-COU interactions on the micronutrient fractions in roots, stems, and leaves. Cu400 significantly reduced the Cu fraction in leaves and the Cu fraction in stems, but it significantly elevated the Cu fraction in roots. The Cu fractions in leaves and stems (roots) increased (decreased) with the increment in COU supply. Cu400 significantly increased or had no significant effects on the Mn fraction in leaves, the Fe fraction in stems, and the Zn, Fe, and Mn fractions in roots, except that Cu400 significantly lowered the Mn fraction in roots at COU0, but it significantly lowered or did not significantly change the Zn and Fe fractions in leaves, the Zn and Mn fractions in stems, and the B fraction in roots. At Cu0.5, the Fe and Mn fractions in leaves and the Zn fraction in roots were significantly higher at COU0 than at COU100, but the Zn fraction in stems and the Fe and Mn fractions in roots were significantly higher at COU100 than at COU0. At Cu400, the Mn fraction in leaves, the Fe and Mn fractions in stems, and the Zn fraction in roots were significantly higher at COU0 than at COU100, but the opposite was the case for the Zn and Fe fractions in leaves, the Zn fraction in stems, and the Mn and Fe fractions in roots.

Figure S2 showed the effects of Cu-COU interactions on the macronutrient fractions in leaves, stems, and roots. Cu400 significantly elevated or did not significantly change the Ca, K, P, and S fractions in leaves, the Ca, K, P, and Mg fractions in stems, and the N and Mg fractions in roots, except that Cu400 significantly decreased the Ca fraction in leaves and the Mg fraction in stems at COU0, but it significantly lowered or did not significantly change the N and Mg fractions in leaves, the N and S fractions in stems, and the S, K, Ca, and S fractions in roots, except that Cu400 significantly elevated the S fraction in roots at COU0. At Cu0.5, the P, Ca, Mg, and S fractions in leaves and the K and S fractions in stems were significantly higher at COU0 than at COU100, but the opposite was the case for the P, K, Ca, Mg, and S fractions in roots. At Cu400, the K fraction in leaves and the N and Ca fractions in roots were significantly higher at COU0 than at COU100, but the opposite was the case for the N and Ca fractions in leaves, the N and Mg fractions in stems, and the K fraction in roots.

### 2.3. Effects of Cu-COU Interactions on Pigments and Gas Exchange in Leaves

**Figure 6:**
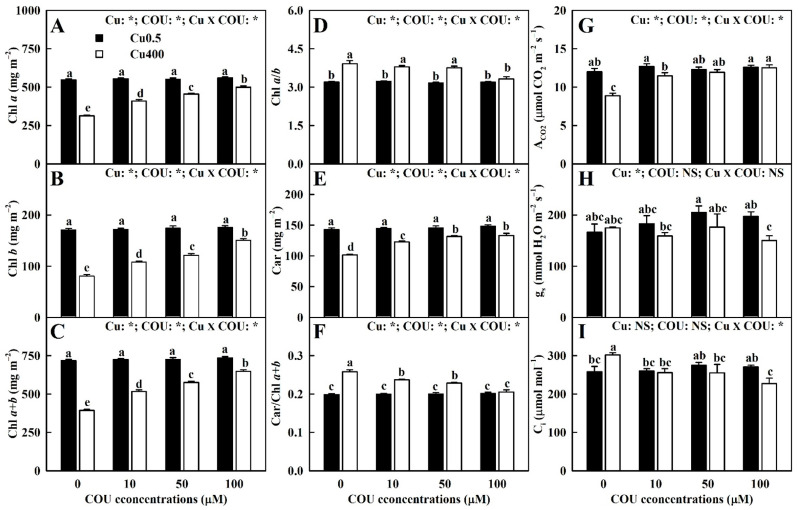
Effects of Cu-COU interactions on the mean (±SE, *n* = 4) Chl *a* (**A**), Chl *b* (**B**), Chl *a+b* (**C**), Chl *a/b* (**D**), Car (**E**), Car/Chl *a+b* (**F**), A_CO2_ (**G**), g_s_ (**H**), and C_i_ (**I**) in leaves. Significant differences were analyzed by two ANOVA and followed by the LSD at *p* < 0.05. Error bars with different letters are significant different at *p* < 0.05. *, significant difference at *p* < 0.05; NS, non-significant difference. A_CO2_, CO_2_ assimilation; Car, carotenoids; Chl, cholorophyll; C_i_, intercellular CO_2_ concentration; g_s_, stomatal conductance.

As shown in Figure 6A–F, the supply of COU mitigated Cu400-induced decreases in the levels of chlorophyll (Chl) *a*, Chl *b*, Chl *a+b*, and carotenoids (Car), as well as Cu400-induced increases in the rations of Chl *a/b* and Car/Chl *a+b*. At Cu400, the Chl *a*, Chl *b*, Chl *a+b*, and Car levels generally elevated with the increase in COU supply, while the Chl *a/b* and Car/Chl *a+b* were significantly lower at COU100 than at COU0, COU10, and COU50. At Cu0.5, the supply of COU did not significantly affect the six parameters.

As shown in Figure 6G–I, the supply of COU alleviated a Cu400-induced decrease in the A_CO2_. Both the g_s_ and C_i_ were not lower at Cu400 than at Cu0.5, except that both the g_s_ and C_i_ in the leaves of COU100-treated seedlings (LCOU100) were significantly higher at Cu0.5 than at Cu400. The supply of COU did not significantly affect these three parameters at Cu0.5 and the g_s_ at Cu400. At Cu400, the leaf A_CO2_ increased with the increments in COU supply, while the reverse was the case for the C_i_.

### 2.4. Effects of Cu-COU Interactions on OJIP Transients and Related Parameters in Leaves

**Figure 7:**
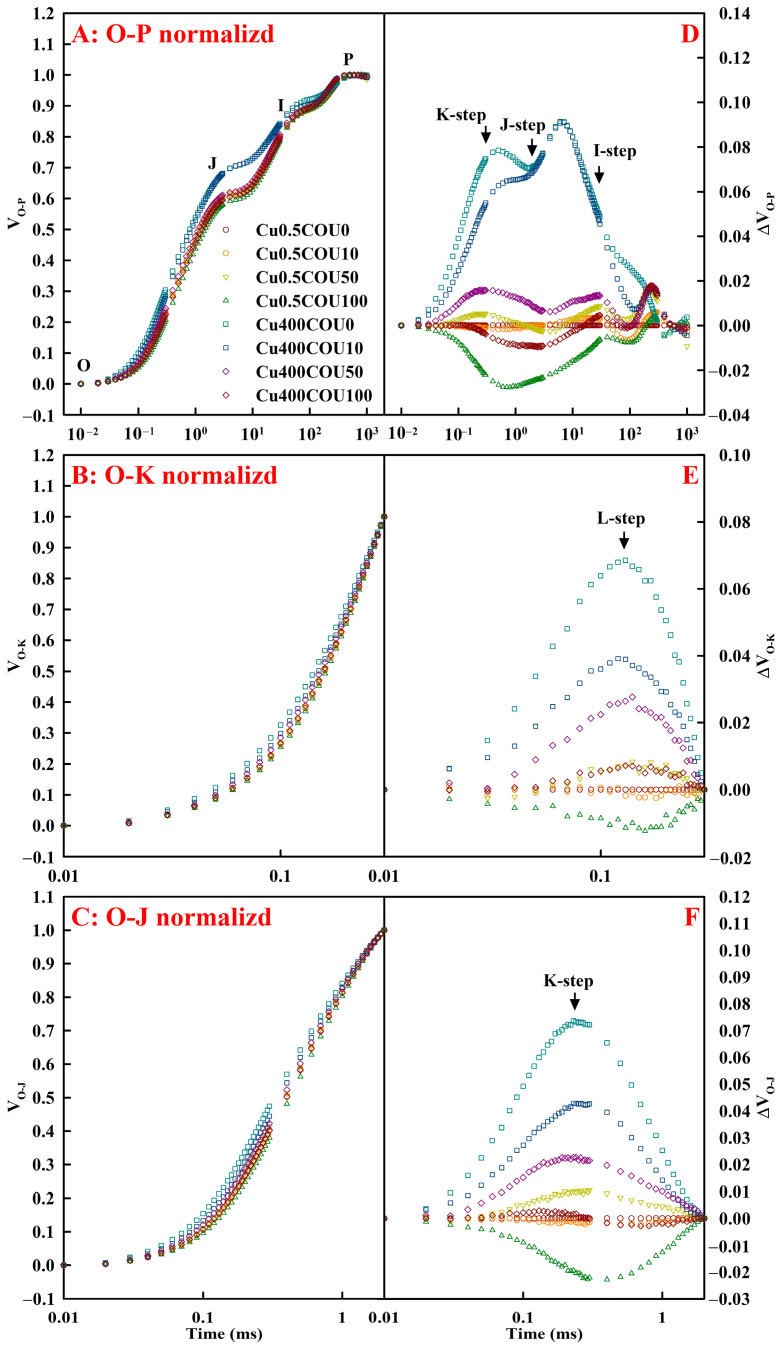
Effects of Cu-COU interactions on the mean OJIP transients of ten measured samples normalized between O-P (V_O-P_), O-K (V_O-K_), and O-J (V_O-J_) (**A**–**C**) and the differences in the eight samples to the reference sample treated with Cu0.5COU0 (**D**–**F**). V_O-P_ = (F_t_ − F_o_)/(F_m_ − F_o_); V_O-K_ = (F_t_ − F_o_)/(F_300μs_ − F_o_); V_O-J_ = (F_t_ − F_o_)/(F_J_ − F_o_); F_m_, maximum fluorescence; F_o_, minimum fluorescence; F_t_, fluorescence intensity at time t after onset of actinic illumination; F_300μs_, fluorescence intensity at 300 μs; F_J_, fluorescence intensity at the J-step (2 ms).

Figure 7 exhibited the impacts of Cu-COU interactions on OJIP transients in leaves. Compared to the OJIP transients in the leaves of Cu0.5COU0-treated seedlings (LCu0.5COU0), the positive ΔI-step (30 ms), ΔJ-step (2 ms), ΔK-step (300 μs), and ΔL-step (~150 μs) in the OJIP transients in the leaves of Cu400-treated seedlings (LCu400) decreased with the increase in COU supply. The OJIP transients in the leaves of Cu0.5-treated seedlings (LCu0.5) exhibited few alterations in response to the COU supply, except that the OJIP transients in LCOU100 had the negative ΔI-, ΔJ-, ΔK-, and ΔL-steps relative to the OJIP transients in the LCu0.5COU0.

**Figure 8:**
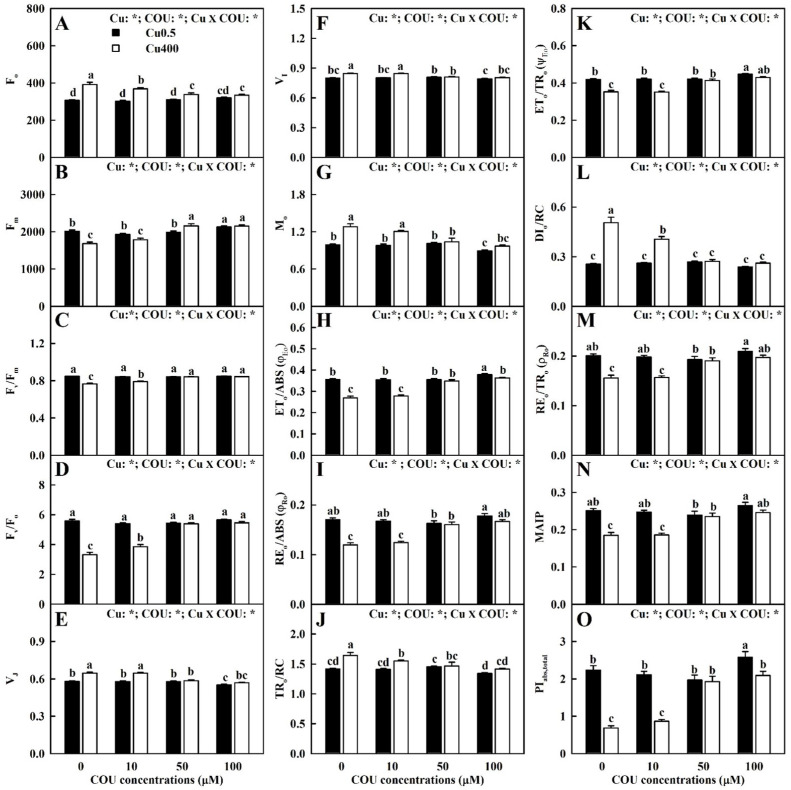
Effects of Cu-COU interactions on the mean (±SE, *n* = 10) F_o_ (**A**), F_m_ (**B**), F_v_/F_m_ (**C**), F_v_/F_o_ (**D**), V_J_ (**E**), V_I_ (**F**), M_o_ (**G**), ET_o_/ABS (**H**), RE_o_/ABS (**I**), TR_o_/RC (**J**), ET_o_/TR_o_ (**K**), DI_o_/RC (**L**), RE_o_/TR_o_ (**M**), MAIP (**N**), and PI_abs,total_ (**O**) in leaves. Significant differences were analyzed by two ANOVA and followed by the LSD at *p* < 0.05. Error bars with different letters are significantly different at *p* < 0.05. *, significant difference at *p* < 0.05. F_o_, minimum fluorescence; F_m_, maximum fluorescence; F_v_/F_m_, maximum quantum yield of primary photochemistry; F_v_/F_o_, maximum primary yield of photochemistry of photosystem II (PSII); V_J_, relative variable fluorescence at the J-step (2 ms); V_I_, relative variable fluorescence at the I-step (30 ms); M_o_, approximated initial slope (in ms^−1^) of the fluorescence transient *V* = *f(t)*; ET_o_/ABS (φ_Eo_), quantum yield for electron transport; RE_o_/ABS (φ_Ro_), quantum yield for the reduction in end acceptors of photosystem I per photon absorbed; TR_o_/RC, trapped energy flux per reaction center; ET_o_/TR_o_ (ψ_Eo_), probability that a trapped exciton moves an electron into the electron transport chain beyond Q_A_^−^; DI_o_/RC, specific energy fluxes per reaction center for energy dissipation; RE_o_/TR_o_ (ρ_Ro_), efficiency with which a trapped exciton can move an electron into the electron transport chain from Q_A_^−^ to the photosystem I end electron acceptors; MAIP, maximum amplitude of IP phase; PI_abs,total_, total performance index.

As shown in Figure 8, Cu400 induced decreases in the F_v_/F_m_, ET_o_/TR_o_, F_m_, F_v_/F_o_, ET_o_/ABS, RE_o_/TR_o_, RE_o_/ABS, MAIP, and PI_abs,total_, and increases in the F_o_, V_I_, V_J_, M_o_, TR_o_/RC, and DI_o_/RC declined with the increment of COU supplementation. Indeed, Cu400 had no significant impacts on these 15 parameters, except that Cu400 induced a significant decrease in ET_o_/ABS and PI_abs,total_ at COU100. At Cu0.5, the supply of COU had no significant impacts on these 15 parameters, except for a significant increase in the F_m_, ET_o_/TR_o_, ET_o_/ABS, RE_o_/TR_o_, MAIP, RE_o_/ABS, and PI_abs,total_ at COU100 and a significant decrease in the V_J_, V_I_, and M_o_, and TR_o_/RC at COU100. At Cu400, the F_o_, V_I_, V_J_, M_o_, TR_o_/RC, and DI_o_/RC declined with the increment of COU supplementation, but the opposite was the case for the other nine parameters.

### 2.5. Effects of Cu-COU Interactions on MDA Levels, HPR, and Antioxidant Enzyme Activities in Leaves and Roots

**Figure 9:**
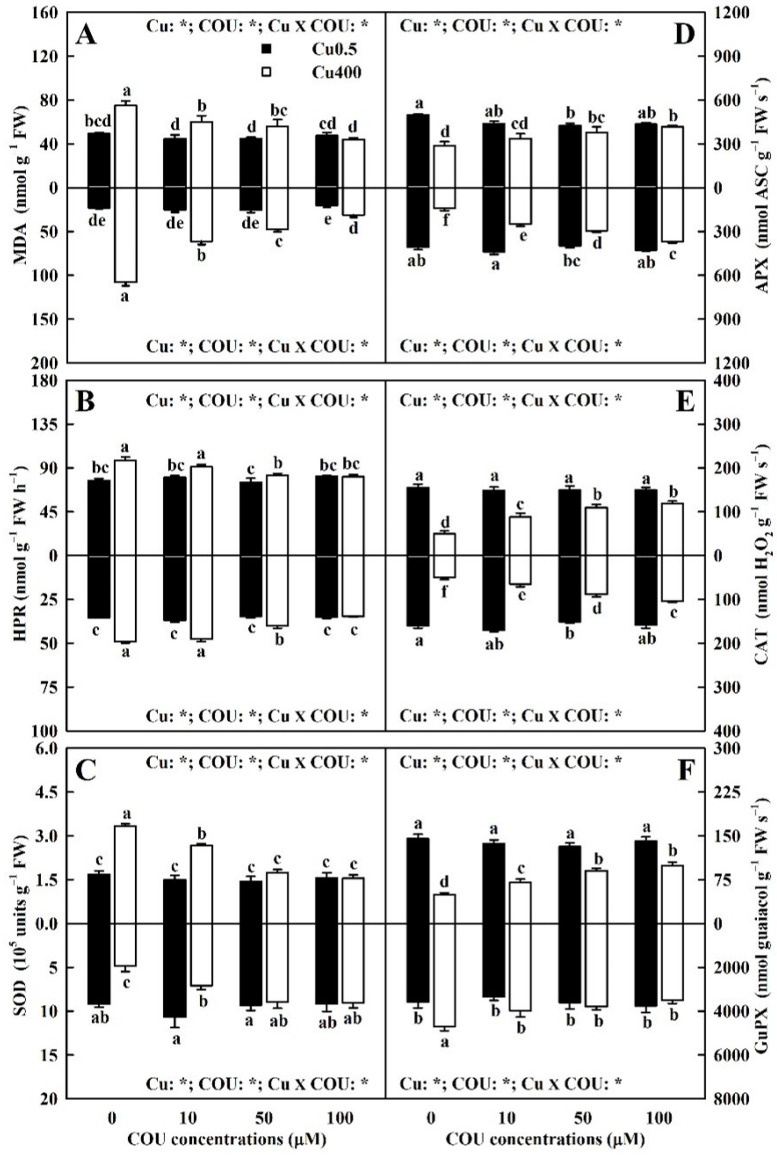
Effects of Cu-COU interactions on the mean (±SE, *n* = 4) concentrations of MDA (**A**), HPR (**B**), and activities of SOD (**C**), APX (**D**), CAT (**E**), and GuPX (**F**) in leaves (above column) and roots (below column). Significant differences were analyzed by two ANOVA and followed by the LSD at *p* < 0.05. Error bars with different letters are significant different at *p* < 0.05. *, significant difference at *p* < 0.05. APX, ascorbate peroxidase; CAT, catalase; COU, coumarin; GuPX, guaiacol peroxidase; HPR, H_2_O_2_ production rate; MDA, malondialdehyde; SOD, superoxide dismutase.

As shown in Figure 9A,B, the supply of COU mitigated Cu400-induced increases in the leaf MDA level, the leaf HPR, and the root HPR. At Cu0.5, the supply of COU did not significantly change the MDA levels and HPR in leaves and roots. At Cu400, the MDA concentrations and HPR in leaves and roots declined with the increment in COU supplementation.

As shown in Figure 9C–F, Cu400 induced increment in the leaf SOD activity and the root GuPX activity and the decrement in the leaf APX, CAT, and GuPX activities, and the root SOD, APX, and CAT activities decreased with the increase in COU supply. At Cu400, the leaf SOD activity and the root GuPX activity decreased with the increase in COU supply, while the leaf APX, CAT, and GuPX activities and the root SOD, APX, and CAT activities increased with the increase in COU supply.

### 2.6. Principal Coordinate Analysis (PCoA) Plots of 21 Parameters for Growth and Fluorescence and 123 Parameters for Nutrients, Pigments, Gas Exchange, MDA, HPR, and Antioxidant Enzymes

**Figure 10:**
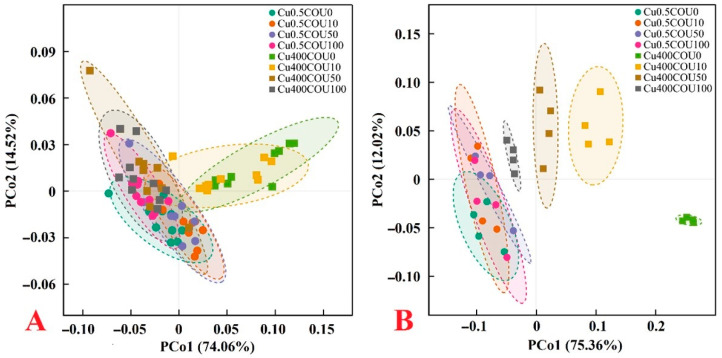
PCoA plots of 21 parameters for growth (6) and fluorescence (15) (**A**) and 123 parameters for nutrients (33 nutrient concentrations, 33 nutrient fractions, 11 nutrient UPR, 11 nutrient UPP, and 14 ratio), pigments (6), gas exchange (3), antioxidant enzymes (8), MDA (2), and HPR (2) (**B**) from *C. sinensis* seedlings submitted to different Cu and COU levels. PCoA, principal coordinate analysis; Cu0.5COU0, 0.5 μM Cu + 0 μM COU; Cu0.5COU10, 0.5 μM Cu + 10 μM COU; Cu0.5COU50, 0.5 μM Cu + 50 μM COU; Cu0.5COU100, 0.5 μM Cu + 100 μM COU; Cu400COU0, 400 μM Cu + 0 μM COU; Cu400COU10, 400 μM Cu + 10 μM COU; Cu400COU50, 400 μM Cu + 50 μM COU; Cu400COU100, 400 μM Cu + 100 μM COU.

For the growth and fluorescence, 0.5 μM Cu + 0 μM COU (Cu0.5COU0), 0.5 μM Cu + 10 μM COU (Cu0.5COU10), 0.5 μM Cu + 50 μM COU (Cu0.5COU50), 0.5 μM Cu + 100 μM COU (Cu0.5COU100), 400 μM Cu + 50 μM COU (Cu400COU50), and 400 μM Cu + 100 μM COU (Cu400COU100) were clustered in the left side. The 400 μM Cu + 10 μM COU (Cu400COU10) were closer to the above six treatments than the 400 μM Cu + 0 μM COU (Cu400COU0) (Figure 10A).

For the nutrients, pigments, gas exchange, MDA, HPR, and antioxidants, the PCo1 could separate the effects of Cu400 on these parameters and the impacts of COU on these parameters in Cu400-treated seedlings, but it could not separate the impacts of COU on these parameters in Cu0.5-treated seedlings. The clustering degree of the four Cu0.5 treatments (Cu0.5COU0, Cu0.5COU10, Cu0.5COU50, and Cu0.5COU100) was higher than that of the four Cu400 treatments (Cu400COU0, Cu400COU10, Cu400COU50, and Cu400COU100). The distance between Cu0.5 and Cu400 declined with the increase in COU supplementation (Figure 10B). Obviously, the supply of COU reduced the impacts of Cu400 on the 144 parameters mentioned above, but Cu400 enhanced the impacts of COU on these parameters.

## 3. Discussion

### 3.1. Copper and COU Show an Interactive Effect on Seedlings

The current results demonstrate that the supply of COU alleviated a Cu400-induced decrease in the seedling growth, increase in the R/S, the yellowing of young leaves, and the decay and death of fibrous roots (Figure 1), as well as Cu400-induced alterations in the OJIP transients (Figure 7) and related parameters (Figure 8), as well as most other physiological parameters (Figure 2, Figure 3, Figure 4, Figure 5, Figure 6 and Figure 9). Cu400 did not significantly affect 75 out of 144 parameters in COU100-treated seedlings, but only 19 out of 144 parameters in COU0-treated seedlings. Also, the changes in these parameters caused by Cu400 were mostly smaller at COU100 than at COU0 (Figure 1, Figure 2, Figure 3, Figure 4, Figure 5, Figure 6, Figure 7, Figure 8 and Figure 9). Growing evidence shows that elevating pH and supplying humic acid, B, P, S, Ca, silicon (Si), and Fe can reduce the levels of Cu in plant tissues and the ability of roots to absorb Cu, thus ameliorating plant Cu toxicity [4,5,8,13,18,35,36,37,38]. The current results indicate that the increase in the Cu UPP (UPR) and the Cu levels in roots, stems, and leaves caused by Cu400 declined with the rise in COU supplementation, and that the Cu levels in the leaves, stems, and roots of Cu400-treated seedlings decreased with the rise in COU supply (Figure 2A,F,K and Figure 4A,L). It is known that COUs can chelate Cu^2+^ to form less mobile non-phytotoxic chelates [39,40]. These results imply that the supply of COU increased Cu chelation by COU and lowered Cu uptake by Cu400-treated seedlings, thereby reducing the accumulation of Cu in roots, stems, and leaves and conferring *C. sinensis* seedling Cu tolerance. The preferential Cu accumulation in the roots of Cu-exposed plants can prevent the uptake of Cu into the sensitive shoots, thereby enhancing the plant’s Cu tolerance [4]. Cu400 increased the Cu fraction in roots more at COU50 and COU100 than at COU0 and COU10, but the Cu fraction in roots of Cu400-treated seedlings (RCu400) and roots of Cu0.5-treated seedlings (RCu0.5) reduced with the rise in COU supply (Figure S1K). Therefore, a COU-mediated reduction in Cu toxicity could not be solely attributed to increased Cu fraction in roots. As shown in Figure 1, Figure 2, Figure 3, Figure 4, Figure 5, Figure 6, Figure 7, Figure 8 and Figure 9, the effects of COU on growth, OJIP transient, and most parameters were greater in seedlings treated with Cu400 than in seedlings treated with Cu0.5. COU100 significantly altered 121 out of 144 parameters in the Cu400-treated seedlings, but only 48 out of 144 parameters in the Cu0.5-treated seedlings. A PCoA indicated that the supply of COU decreased the effects of Cu400 on the 144 parameters, and Cu400 intensified the effects of COU on the 144 parameters (Figure 10). The current results show that Cu and COU had an interactive impact on 99 out of 144 parameters (Figure 1, Figure 2, Figure 3, Figure 4, Figure 5, Figure 6, Figure 8 and Figure 9). Obviously, Cu and COU exhibited an interactive effect on citrus seedlings.

### 3.2. Coumarin Reduced Oxidative Injury in Leaves and Roots Caused by Excessive Cu

**Figure 11:**
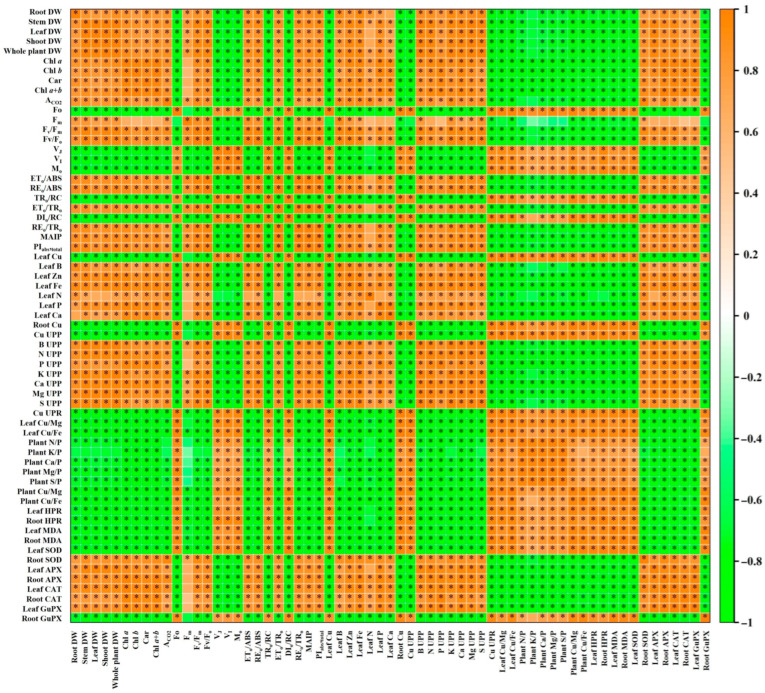
Matrices of Pearson correlation coefficients (PCCs) for the mean values of 63 physiological parameters in *C. sinensis* leaves and roots. Data came from Figure 1, Figure 2, Figure 3, Figure 4, Figure 5 and Figure 6, Figure 8, and Figure 9. *, significant difference at *p* < 0.05; leaf element (pigment), leaf (pigment) concentration; root element, root element concentration; leaf (root) enzyme, leaf (root) enzyme activity.

Copper can stimulate ROS formation through the Haber–Weiss and Fenton reactions [41]. The current findings indicated that Cu400 led to an increase in excess energy excitation (EEE) in leaves due to decreased A_CO2_ (Figure 6A), as shown by the elevated DI_o_/RC (Figure 8L). The increased EEE can potentially cause the formation of ROS [42]. As shown, Cu400 increased the MDA accumulation and HPR in leaves and roots, with a greater increment in roots than in leaves (Figure 9A,B). This might be related to the accumulation of most Cu in RCu400 (Figure 2A,F,K) [8]. As shown in Figure 11 and Table S1, a positive correlation existed between any two parameters of MDA concentration, HPR, Cu concentration, Cu UPP, and Cu UPR. These results suggest that Cu400 increased the Cu absorption and Cu levels in leaves and roots, thereby inducing the ROS formation and over-accumulation and causing oxidative injury in leaves and roots.

Reactive oxygen species can be scavenged through antioxidant enzymes, which are the first line of defense against oxidative stress [43]. O_2_^−^ is often the first ROS yielded in plant tissues. The O_2_^−^ yielded is then dismutated to H_2_O_2_ and O_2_ by SOD [44]. The H_2_O_2_ formed by SOD can be converted into H_2_O through H_2_O_2_-scavenging enzymes such as CAT, APX, and peroxidase [45]. The current results indicate that at COU0, Cu400 significantly elevated and reduced the SOD activities in leaves and roots, respectively (Figure 9C). This agrees with the results obtained on *C. sinensis* leaves and roots [18,41]. Excessive Cu inhibited the SOD activity in sunflower roots [46]. The SOD activity in tomato leaves was induced early after exposure to 25 μM CuSO_4_, reached its maximum activity after 12 h, and then decreased, but kept slightly higher than the control until the end of the experiment (96 h) [47]. Thounaojam et al. [48] observed that after the first day of treatment, SOD activities only elevated in the shoots and roots of rice seedlings treated with 100 μM Cu, while after the fifth day of treatment, SOD activities increased in the shoots of seedlings treated with 10, 50, and 100 μM Cu, as well as in the roots of seedlings treated with 50 and 100 µM Cu. However, excessive Cu led to a decrease in SOD activities in the leaves of bean [49] and *Withania somnifera* [50]. Excessive Cu increased the SOD activities in the leaves and roots of rice [21] and *Astragalus neo-mobayenii* [51], as well as the leaves of mulberry [52].

Catalase can directly catalyze the conversion of H_2_O_2_ into H_2_O and O_2_ in peroxisome and mitochondrion [42], and is essential for detoxifying ROS under stress conditions [44]. The key pathway for the detoxification of H_2_O_2_ in chloroplast is the ASC-glutathione cycle, as CAT does not exist in chloroplast. In this pathway, APX utilizes ASC as the electron donor to reduce H_2_O_2_ to H_2_O [42,53]. As shown in Figure 9D,E, at COU0, Cu400 significantly reduced the CAT and APX activities in leaves and roots. This agreed with the results obtained by Zhang et al. [41] and Chen et al. [18] on *C. sinensis*. In rice, Mostofa and Fujita [21] reported that 75 and 150 μM Cu increased and decreased CAT activity in leaves, respectively, but they decreased CAT activity in roots, and that 75 and 150 μM Cu did not alter and increased its activity in leaves, respectively, but they elevated its activity in roots. Thounaojam et al. [48] indicated that the APX activities in rice leaves and roots elevated progressively in a time- and dose-dependent manner, but Cu concentrations and treatment duration did not change the CAT activity. Excessive Cu elevated the CAT and APX activities in mulberry leaves [52]. In tomato, Mazhoudi et al. [54] showed that Cu excess lowered CAT activity in roots and did not alter its activities in stems and leaves, but it decreased APX activity in leaves and did not change its activities in roots and stems.

Also, peroxidase plays a key role in the removal of H_2_O_2_ in plants [55]. The current results indicate that, at COU0, Cu400 significantly decreased (increased) the GuPX activity in leaves (roots) (Figure 9F). This agrees with the results reported on *C. sinensis* [18,41]. Martins and Mourato [56] found that at 50 μM Cu, the GuPX activity in tomato leaves began to increase two days after Cu treatment, reached its highest activity after three days, and then decreased over time; at 100 μM Cu, the GuPX activity increased one day after Cu treatment, reached its highest activity after three days, and then decreased over time, and at 200 and 350 μM Cu, the GuPX activity increased one day after Cu treatment and then decreased over time. The GuPX activity was lower in the leaves of 200 and 350 μM Cu-treated seedlings than in the control after six days, but not in the leaves of 50 and 100 μM Cu-treated seedlings. In tomato, excessive Cu stimulated the GuPX activities in roots and stems, but not in leaves [54]. The GuPX activities in rice and *A. neo-mobayenii* leaves and roots increased with an increment in Cu concentrations [48,51].

Collectively, these findings suggest that the impacts of Cu on SOD, CAT, APX, and GuPX activities depended on Cu concentration, Cu exposure time, plant species, and tissues.

Growing evidence shows that increasing pH [41] and exogenous applications of B [18], salicylic acid [21], melatonin [57], reduced glutathione (GSH), sodium nitroprusside [16], acetylsalicylic acid [58], H_2_S [59], and ʟ-glutamic acid [3] can protect plants from oxidative injury by lowering the formation of ROS stimulated by Cu toxicity and by preventing Cu toxicity from damaging the ROS detoxification system, thereby reducing Cu toxicity in plants. Shad et al. [22] suggested that COU-mediated amelioration of sesame Mn toxicity was ascribed to decreased oxidative injury due to downregulated ROS generation and upregulated ROS scavenging system. The current results indicate that the supplementation of COU alleviated Cu400-induced increase in the MDA concentrations and HPR, as well as changes in antioxidant enzyme activities in leaves and roots (Figure 9). Regression analysis indicated that MDA (HPR) was negatively related to APX and CAT in leaves and roots, SOD in roots, and GuPX in leaves, and positively related to SOD in leaves and GuPX in roots (Figure 11 and Table S1). These results suggest that the supply of COU reduced Cu400-induced ROS (H_2_O_2_) formation and the impairment of Cu400 on antioxidant enzymes in leaves and roots, thereby enhancing their ability to maintain a balance between ROS formation and scavenging, and hence alleviating their Cu toxicity.

### 3.3. The Promoting Effects of COU on the Growth of Cu-Exposed Seedlings

The current results show a significant alleviation of COU supply on Cu400-induced growth decline in seedlings (Figure 1). The growth of plants depends on the carbohydrates provided by photosynthesis [60]. It was found that a positive relationship existed between any two parameters of whole plant DW, shoot DW, root DW, Car, Chl *a+b*, and A_CO2_ (Figure 11 and Table S1). Therefore, the supply of COU reduced the impacts of Cu400 on photosynthesis, thereby promoting seedling growth at Cu400.

The functional injury and growth reduction caused by Cu toxicity usually occur earlier in the roots than in the aboveground parts, as Cu preferentially accumulates in the roots of plants exposed to Cu [61]. The earlier root damage and reduced growth can in turn affect nutrient absorption, thus inhibiting plant growth [14]. The decay and death of fibrous roots occurred in the Cu400COU0-treated seedlings, but not in the Cu400COU100-treated seedlings (Figure 1H). Regression analysis showed that whole plant DW (Cu UPP and UPR) was positively (negatively) related with Ca, Mg, K, P, N, S, and B UPP and UPR, except for the relationship between Cu UPP and S UPR (*r* = −0.6767), and that the whole plant, shoot, and root DW were negatively related with Cu UPP and UPR (Figure 11 and Table S1). Previous reports indicate that the supply of B, Ca, P, S, and Mg could alleviate excessive Cu-induced inhibition of plant growth [5,17,35,38,62], and that COU-mediated mitigation of sesame growth decline caused by Mn toxicity was ascribed to improved nutrient acquisition [22]. These findings suggest that the supply of COU reduced Cu UPP and UPR and alleviated root damage caused by Cu400, thereby enhancing B, N, Ca, K, P, Mg, and S UPP, and hence promoting the growth of Cu400-treated seedlings.

Evidence shows that Cu toxicity can lead to nutritional imbalances in plants and constrain their growth [4]. Cu has been suggested to have an antagonistic action on P. Studies have shown that P starvation is the key limiting factor for plant growth exposed to Cu toxicity [4,5,63]. As expected, Cu400 was more effective in reducing P UPP than the other five macronutrient UPPs, thereby elevating the plant N/P, Ca/P, K/P, Mg/P, and S/P. At Cu400, these ratios decreased with the rise in COU supplementation (Figure 4F–K and Figure 5H–L). It was found that individual deficiency of nitrate or phosphate (Pi) in chickpea leaves and roots caused a greater molecular response than jointed deficiency of nitrate and Pi [64]. Also, Cu has an antagonistic impact on Fe [49] and Mg [65]. As expected, Cu400 increased the plant Cu/Mg and Cu/Fe, especially at COU0 (Figure 5M,N). It was found that the whole plant DW was negatively related with the plant Ca/P, Mg/P, N/P, S/P, Cu/Mg, and Cu/Fe, and displayed a decreasing trend with the rise in plant K/P (*r* = −0.7060) (Figure 11 and Table S1). These findings suggest that the supply of COU alleviated the damage of Cu400 to nutrient homeostasis and balance, thereby promoting the growth of seedlings treated with Cu400, and that Cu400-induced P starvation might function in Cu400-induced reduction in seedling growth.

As is well known, the accumulation of ROS can lead to oxidative stress, damage important cellular components (proteins, DNA, and lipids), and ultimately hinder plant growth [66]. Pre-treatment of *Brassica juncea* seeds with castasterone (a C-28 brassinosteroid) increased seedling growth by lowering the ROS levels under Cu excess [67]. The current results indicate that the Cu400-induced increase in MDA concentrations and HPR declined with the rise in COU supply (Figure 9A,B), and root (leaf) DW was negatively and significantly related to root (leaf) MDA and HPR (Figure 11 and Table S1). Therefore, the supply of COU mitigated the growth reduction caused by Cu400 by reducing oxidative damage.

### 3.4. The Supply of COU Mitigated the Leaf Pigment Reduction Caused by Cu Toxicity

The current results indicate that Chl *a+b*, Chl *a,* Chl *b*, and Car levels are negatively related to the Cu level in leaves (Figure 11 and Table S1), and the decrease in leaf pigment concentrations caused by Cu400 decline with the rise in COU supplementation (Figure 9A–C,E). Chloroplast, the major site of ROS formation under stress conditions, is the target of ROS-triggered damage [68]. The oxidative damage caused by excessive ROS can cause a decrement in pigment level [69]. Evidence shows that Cu^2+^ competes with Mg^2+^ for binding sites on root surfaces, thereby inhibiting Mg^2+^ uptake [70]. Cu^2+^ can substitute Mg^2+^ in Chl molecules [1] and damage the chloroplast’s ultrastructure [71], leading to a decreased concentration of photosynthetic pigments [17]. In *Ceratophyllum demersum*, excessive Cu firstly disrupted the light-harvesting complex of photosystem II (PSII), where Cu^2+^ replaced Mg^2+^ [72]. The current results show that the reduction in leaf F_v_/F_o_ (an indicator of structural injury to thylakoid) [73] and increase in leaf Cu/Mg, HPR, and MDA concentration caused by Cu400 decreased with the rise in COU supplementation (Figure 5F, Figure 8D, and Figure 9A,B). Our regression analysis indicated that the Chl *a+b*, Chl *a,* Chl *b*, and Car levels were negatively related to the leaf Cu/Mg, HPR, and MDA level, but positively related to F_v_/F_o_ (Figure 11 and Table S1). These findings suggest that the supply of COU reduced the substitution of Mg^2+^ in Chl molecules by Cu^2+^ and the (oxidative) damage of chloroplast caused by Cu400, thereby lowering pigment decline caused by Cu400.

Previous reports indicate that excessive Cu-induced decline of pigments is due to excessive Cu-induced Fe deficiency [12], and that excessive Cu lowers the Chl *a+b* concentration in bean leaves through competition with Fe^2+^ [49]. It was observed that the reduction in leaf Fe level and the increase in leaf Cu/Fe caused by Cu400 declined with the rise in COU supplementation (Figure 2D and Figure 5G), and the Chl *a+b*, Chl *a,* Chl *b*, and Car levels were positively (negatively) related with the leaf Fe level (Cu/Fe) (Figure 11 and Table S1). Therefore, the supply of COU might reduce the Cu400-induced decrement in leaf Fe level and increment in leaf Cu/Mg, as well as the competition of Cu^2+^ with Fe^2+^, thereby alleviating the leaf pigment decline caused by Cu400.

The deficiency of other nutrients (P, N, Ca, Zn, and B) can also lower the photosynthetic pigments in leaves [2,74,75,76,77,78]. The current results indicate that the supply of COU mitigated the decrease in the B, Zn, N, P, and Ca levels in leaves (Figure 2B,C and Figure 3A,B,D), as well as the B, N, P, K, Ca, and S UPP caused by Cu400 (Figure 4B,F–K). Our regression analysis indicated that the leaf Chl *a+b*, Chl *a,* Chl *b*, and Car levels were positively related with the leaf B, Zn, N, P, and Ca levels, as well as the B, N, P, K, Ca, and S UPP (Figure 11 and Table S1). These results suggest that the supply of COU elevated the nutrient uptake in Cu400-treated seedlings, thereby alleviating Cu400-induced pigment decline in leaves.

### 3.5. The Supply of COU Mitigated the Leaf A_CO2_ Decline Caused by Cu Toxicity

The current results indicate that the reduction in leaf A_CO2_ caused by Cu400 and the COU-mediated alleviation of leaf A_CO2_ decline caused by Cu400 (Figure 6G) could not be explained by stomatal limitation, as the Cu-COU interactions had no significant effects on both the g_s_ and C_i_, except for a decrease in g_s_ and C_i_ at Cu400COU100 and an increase in g_s_ at Cu400COU0 (Figure 6H,I).

Previous research showed that damage to the entire photosynthetic electron transport chain (PETC) from the PSII donor side to the reduction in PSI end electron acceptors was the main reason for the decline of A_CO2_ in leaves of Cu-exposed plants [15]. As is well known, Cu excess makes PSII sensitive to photoinhibition, as it can outcompete Fe, causing a reduction in Chl concentration in leaves [49]. Under strong light, the [Cu]-Chl yielded mainly in the pheophytin *a* of PSII reaction center can cause the entire photosystem to lose function [72]. The replacement of Chl Mg^2+^ by Cu^2+^ is believed to be a damaging mechanism, causing a decrease in photosynthesis [1]. As expected, photo-inhibitory damage occurred in the LCu400 [8,79], as indicated by the declined F_v_/F_m_ and ET_o_/ABS, the elevated DI_o_/RC (Figure 8D,H,L), and the altered OJIP transients in leaves (Figure 7). The photo-inhibitory damage in the LCu400 was alleviated by the supply of COU.

This study indicates that the supply of COU mitigated the positive ΔI-, ΔJ-, ΔK-, and ΔL-step (Figure 7) and the reduction in MAIP (Figure 8N) in leaves caused by Cu400. The positive ΔL-step in the LCu400 suggested that Cu400 caused a loss in the stability of the PSII units and a decrease in the energy exchange between independent PSII units [80]. The positive ΔK-step implied that, due to the damage of oxygen-evolving complex (OEC) in LCu400, the donation of electrons from OEC to the oxidized PSII reaction center became limited [81]. The Cu400-induced increase in V_J_ and V_I_ and decrease in MAIP suggested that Cu400 damaged the PSII acceptor side more than the PSII donor side [82]. This was consistent with a reduction in F_v_ and an increment in F_o_, which is a characteristic of photo-inhibitory injury in the PSII acceptor side [83]. The positive ΔI-, ΔJ-, ΔK-, and ΔL-steps have been obtained on leaves of aluminum (Al)- and Mn-treated *C. sinensis* seedlings [84,85]. It was found that Cu400-induced reduction in leaf PI_abs,total_, MAIP, RE_o_/ABS, and RE_o_/TR_o_ reduced with the rise in COU addition (Figure 8I,M–O), demonstrating that the supply of COU alleviated the damage of Cu400 to the reduction in PSI end-electron acceptors. These findings suggest that the supply of COU mitigated the damage of Cu400 to the whole PETC.

The production of ROS driven by light can lead to oxidative injury of key photosynthetic components, thus repressing photosynthesis [86]. As shown in Figure 9A,B, the supply of COU reduced the formation of ROS and oxidative injury in leaves caused by Cu400. Our regression analysis shows that a negative or positive correlation existed between any two parameters of Cu concentration, Cu/Fe, Cu/Mg, HPR, MDA concentration, Car concentration, Chl *a+b* concentration, A_CO2_, F_v_/F_m_, F_o_, F_m_, F_v_/F_o_, V_I_, V_J_, M_o_, RE_o_/ABS, TR_o_/RC, ET_o_/TR_o_, ET_o_/ABS, DI_o_/RC, MAIP, RE_o_/TR_o_, and PI_abs,total_ (Figure 11 and Table S1).

Except for Mg and Fe, the deficiency of other nutrients (B, Zn, N, P, and Ca) also lower A_CO2_ and damage the PETC in leaves [77,81]. It was found that the P, N, B, Ca, and Zn levels were positively related to A_CO2_, F_v_/F_m_, F_m_, F_v_/F_o_, ET_o_/ABS, RE_o_/ABS, ET_o_/TR_o_, RE_o_/TR_o_, PI_abs,total_, and MAIP, but negatively related to M_o_, F_o_, V_I_, V_J_, TR_o_/RC, and DI_o_/RC in leaves, except for the N concentration in relation to V_J_ (*r* = −0.6706), V_I_ (*r* = −0.6983), ET_o_/ABS (*r* = 0.7056), ET_o_/TR_o_ (*r* = 0.6707), RE_o_/TR_o_ (*r* = 0.6985), and MAIP (*r* = 0.6964) and the F_m_ in relation to N concentration (*r* = 0.5608), P concentration (*r* = 0.6735), and Ca concentration (*r* = 0.6529) (Figure 11 and Table S1).

Considered together, the supply of COU reduced the Cu concentration, competition Cu^2+^ with Fe^2+^ and Mg^2+^, and oxidative damage, and improved the pigment level and nutrient status, thereby mitigating the photo-inhibitory damage of the entire PETC and A_CO2_ decline in leaves caused by Cu400.

## 4. Materials and Methods

### 4.1. Seedling Culture and Treatments

Seedling culture and Cu-COU treatments were performed according to Huang et al. [8], with some modifications. Humic acid was a treatment factor in the previous report and was replaced by COU in this study. ‘Xuegan’ (*Citrus sinensis* (L.) Osbeck) seeds were germinated in plastic seedling trays containing sand. Six weeks after germination, uniform seedlings were grown in 6 L pots (two seedlings per pot) containing sand in a greenhouse under natural light, temperature, and relative humidity at Fujian Agriculture and Forestry University. Seven weeks after transplantation, each pot was irrigated with nutrient solution six times a week until some solution began to flow out of the bottom hole of the pot (~500 mL). The formula of the nutrient solution was as follows: 2.5 mM Ca(NO_3_)_2_, 0.5 mM KH_2_PO_4_, 2.5 mM KNO_3_, 1 mM MgSO_4_, 20 μM Fe-EDTA, 10 μM H_3_BO_3_, 2 μM ZnSO_4_, 2 μM MnCl_2_, 0.5 μM CuCl_2_, 0.065 μM (NH_4_)_6_Mo_7_O_24_, 0.5 (control or Cu0.5) or 400 (Cu toxicity, Cu excess or Cu400) μM CuCl_2_, and 0 (COU0), 10 (COU10), 50 (COU50), or 100 (COU100) μM COU. The Cu concentrations were chosen according to the study performed by Li et al. [15], who examined the impacts of 0.5, 100, 200, 300, 400, and 500 μM CuCl_2_ on the growth and related parameters in ‘Xuegan’ seedlings. The COU concentrations were chosen according to the studies by Hui [31] and Parvin et al. [33]. To prevent Cu precipitation, adjust the pH of the solution to 4.8 with HCl. There were 8 treatments, each with 12 pots in a completely randomized design. Twenty-four weeks after Cu-COU treatments, about 5 mm in length of white root tips and the recently fully expanded (about 7-week-old) leaves were used for all measurements, except for nutrients. On a sunny noon, leaf disks with a diameter of 6 mm and about 5 mm in length of white root tips were taken and immediately frozen in liquid N_2_, and then stored at −80 °C until enzymes and metabolites were extracted. HPR was determined using fresh leaves and roots. These un-sampled seedlings were used to measure nutrients and biomass.

### 4.2. Measurements of Biomass and Leaf Pigments

Ten seedlings per treatment from different pots were taken and divided into roots, stems, and leaves after they were washed thoroughly with tap water. After drying to a constant weight at 70 °C, their DW was weighed [5].

Leaf Chl *a*, Chl *b*, and Car concentrations were assayed according to Lichtenthaler [87] after extraction with 80% (*v*/*v*) acetone.

### 4.3. Measurements of Gas Exchange, OJIP Transients, and Calculations of Fluorescence Parameters in Leaves

Leaf A_CO2_, g_s_, and C_i_ were measured with a CIRAS-2 portable photosynthesis system (PP System, Herts, UK) between 9:00 and 12:00 a.m. on a sunny day at a controlled CO_2_ concentration of ~400 μmol mol^−1^, a leaf temperature of ~22 °C, and a controlled light intensity of ~1000 μmol m^−2^s^−1^ [5].

Leaf OJIP transients were made with a Handy PEA (Hansatech Instruments Limited, Norfolk, UK) after seedlings were dark-adapted for 3 h at room temperature (~25 °C). All fluorescence parameters were calculated according to Jiang et al. [84] and Kalaji et al. [81]. Table S2 lists the parameters, formulae, and their descriptions using data extracted from OJIP transients.

### 4.4. Analysis of Nutrients in Leaves, Stems, and Roots

The middle sections of stems, fibrous roots, and recently fully expanded leaves were collected for the analysis of nutrients [88]. The samples were first washed in 0.2% HCl (~30 s), then rinsed in tap water, and finally washed in distilled water. After being wiped with towel, samples were first oven-dried at 105 °C for 30 min, then at 65 °C until constant weight (48–72 h), ground, and stored for analysis [11]. Mg, Ca, Zn, Fe, Mn, Cu, K, P, S, B, and N were measured according to Huang et al. [8]. Briefly, for the assays of K, S, P, Zn, Fe, Mg, Ca, Mn, and Cu, 0.2 g root (leaf) samples or 0.4 g stem samples were digested in 6 mL mixture of HNO_3_/HClO_4_ (5/1; *v*/*v*). K was determined with a FP640 Flame Photometry (Shanghai Precision Scientific Instrument Co., Ltd., Shanghai, China). S was measured using the simple turbidimetric method based on the formation of BaSO_4_ precipitate in colloid form. P was determined by colorimetrically as blue molybdate–phosphate complexes. Zn, Fe, Mg, Ca, Mn, and Cu were measured using a PinAAcle 900F Atomic Absorption Spectrometer (Perkinelmer Singapore Pte Ltd., Singapore). After ashing the sample at 500 °C for 5 h and dissolving it in 0.1 M HCl, B in the solution was determined by the curcumin method. N was measured by indophenol blue spectrophotometry (Forestry Industry Standards of the People’s Republic of China; LY/T 1269-1999 [89]). UPP was the sum of the element content (element concentration × tissue DW) in the leaves, stems, and roots. UPR was calculated as the sum of element content in leaves, stems, and roots/root DW. The nutrient fractions were calculated as described by Huang et al. [74].

### 4.5. Analysis of MDA Concentrations, HPR, and Antioxidant Enzyme Activities in Leaves and Roots

Leaf and root HPR were assayed by the reduction in nitroblue tetrazolium (NBT) [90]. Leaf and root MDA concentrations were estimated using the modified thiobarbituric acid-reactive substances after extraction with 80% (*v*/*v*) ethanol [91].

For the analysis of APX, GuPX, CAT, and SOD activities, ~30 mg frozen leaf disks (0.6 cm in diameter) and root samples were homogenized with 2 mL of 50 mM KH_2_PO_4_-KOH (pH 7.5), 1 mM disodium ethylendiamine tetraacetate (EDTA-Na_2_), 5% (*w*/*v*) insoluble polyvinylpolypyrrolidone (PVPP), and 0.5% (*w*/*v*) Triton X-100. After centrifuging at 13,000× *g* and 4 °C for 10 min, the extract was used for the analysis of enzyme activities. APX activity was determined at 290 nm in a mixture (1 mL) containing 50 mM HEPES-KOH (pH 7.6), 0.5 mM ASC, 0.1 mM EDTA-Na_2_, 0.2 mM H_2_O_2_, and 50 μL of extract [92]. CAT activity was assayed by following the decrease in absorbance at 240 nm in a mixture (1 mL) containing 100 mM potassium phosphate buffer (pH 7.0), 10 μL of 10% (*w*/*v*) H_2_O_2_, and 10 μL of extract [93]. GuPX activity was determined at 470 nm in a reaction mixture (1 mL) containing 100 mM potassium phosphate buffer (pH 6.0), 5 μL of 10% (*w*/*v*) H_2_O_2_, 16 mM guaiacol, and 20 μL of extract [93]. SOD activity was assayed at 560 nm in a mixture containing methionine, riboflavin, NBT, and extract [94].

### 4.6. Statistical Analysis

Results were the mean ± SE (*n* = 10 for biomass and fluorescence parameters or 4 for the other parameters), except for the mean OJIP transients. Significant differences were analyzed by four (COU levels) × two (Cu levels) ANOVA and followed by the LSD at *p* < 0.05 using DPS 7.05 (Hangzhou RuiFeng Information Technology Co., Ltd., Hangzhou, China). PCoA was carried out using ChiPlot (https://www.chiplot.online/, accessed on 5 June 2024).

## 5. Conclusions

**Figure 12:**
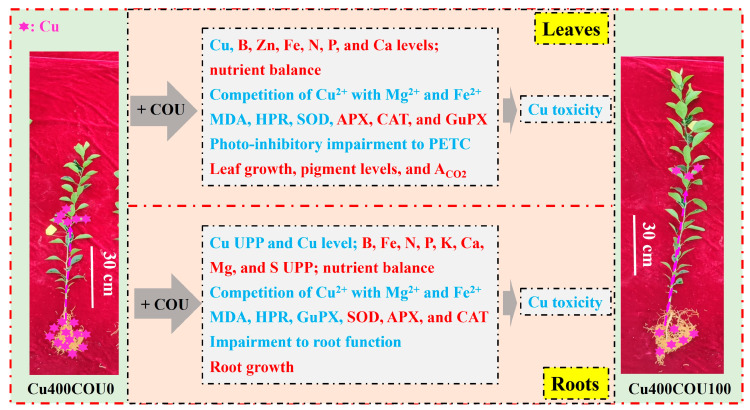
A proposed model for the underlying mechanisms by which COU mitigated copper toxicity in *Citrus sinensis* seedlings. Red, upregulation. Blue, downregulation.

The current study demonstrates that COU addition mitigated Cu400-induced increase in Cu uptake and oxidative stress in roots and leaves, decrease in growth, nutrient uptake, and leaf pigment concentrations and A_CO2_, and photo-inhibitory impairment to the whole PETC. Further analysis indicated that the COU-mediated improvement of nutrient status and mitigation of oxidative stress might contribute to enhanced leaf pigment levels, A_CO2_, and growth of Cu400-treated seedlings by lowering the damage of Cu400 to roots and leaves (chloroplast ultrastructure and PETC) (Figure 12). The current findings supported the hypothesis that exogenous application of COU reduced the inhibitory action of excessive Cu on seedling growth through reducing Cu absorption and oxidative injury and improving plant nutrient status (homeostasis and balance) and photosynthetic performance. This study provides new evidence on the mechanism for the COU-mediated mitigation of Cu toxicity in plants and lays the foundation for further research on the molecular mechanisms of Cu toxicity alleviation mediated by COU in plants.

## Data Availability

Data are archived in L.-S.C.’s lab and are available upon request.

## Supplementary Materials

The following supporting information can be downloaded at: https://www.mdpi.com/article/10.3390/plants13243584/s1, Figure S1: Effects of Cu-COU interactions on the mean (±SE, *n* = 4) micronutrient fractions in leaves (A–E), stems (F–J), and roots (K–O); Figure S2: Effects of Cu-COU interactions on the mean (±SE, *n* = 4) macronutrient fractions in leaves (A–F), stems (G–L), and roots (M–R); Table S1: Pearson correlation coefficient matrix for the mean values of 144 physiological parameters in eight Cu and COU treatment combinations; Table S2: Summary of parameters, formulae and their description using data extracted from chlorophyll (Chl) *a* (OJIP) transient.
