# Supplementary material for: The Ameliorative Effect of Coumarin on Copper Toxicity in Citrus sinensis: Insights from Growth, Nutrient Uptake, Oxidative Damage, and Photosynthetic Performance
Source: Plants (Basel). 2024 Dec 22;13(24):3584. doi: 10.3390/plants13243584 (PMC11678025; doi:10.3390/plants13243584)
Supplement: Supplementary file 1 [file plants-13-03584-s001.zip › 2024HuangPlantsFigures S1-S2.pdf]

Supplementary Figures S1-S2

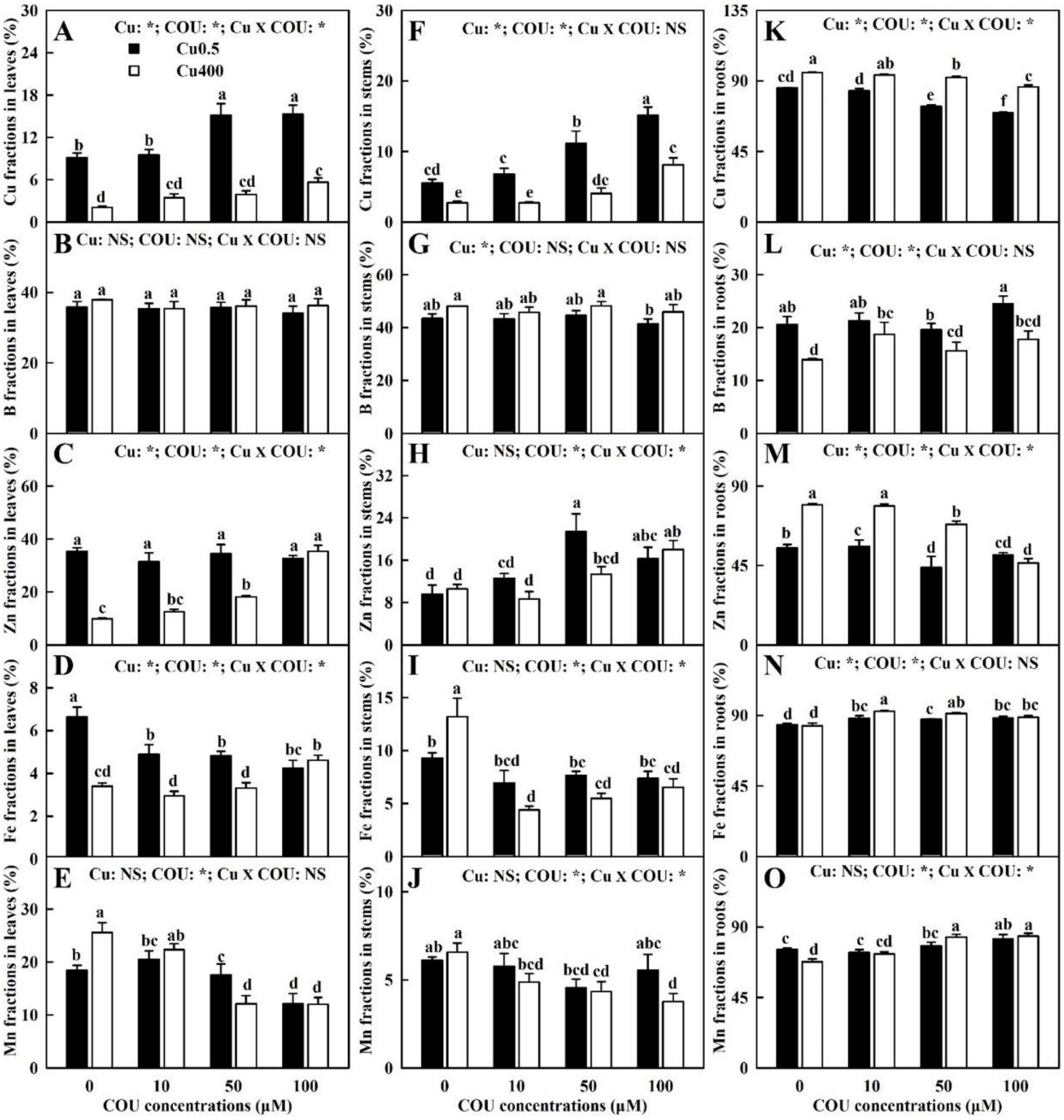

Figure S1. Effects of Cu-COU interactions on the mean (± SE, *n* = 4) micronutrient fractions in leaves (**A-E**), stems (**F-J**), and roots (**K-O**).

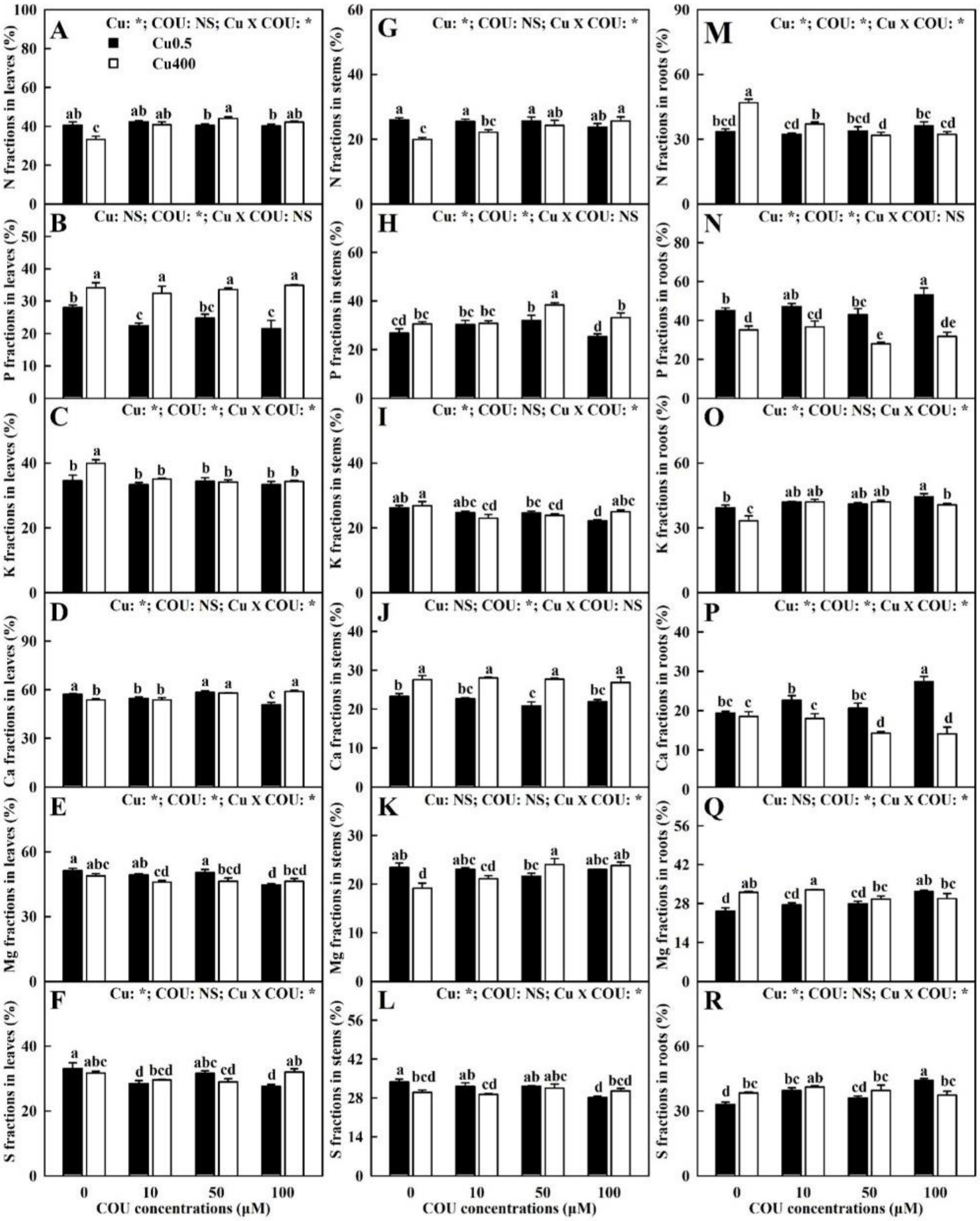

Figure S2. Effects of Cu-COU interactions on the mean (± SE, *n* = 4) macronutrient fractions in leaves (**A-F**), stems (**G-L**), and roots (**M-R**).
