# Supplementary material for: The Ameliorative Effect of Coumarin on Copper Toxicity in Citrus sinensis: Insights from Growth, Nutrient Uptake, Oxidative Damage, and Photosynthetic Performance
Source: Plants (Basel). 2024 Dec 22;13(24):3584. doi: 10.3390/plants13243584 (PMC11678025; doi:10.3390/plants13243584)
Supplement: Supplementary file 1 [file plants-13-03584-s001.zip › 2024HuangTable S2.pdf]

Table S2. Summary of parameters, formulae and their description using data extracted from chlorophyll (Chl) *a* (OJIP) transient.

F_o	Minimum fluorescence, when all PSII RCs are open
F_m	Maximum fluorescence, when all PSII RCs are closed
F_J	Fluorescence intensity at the J-step (2 ms)
F_I	Fluorescence intensity at the I-step (30 ms)
$V_J = (F_{2ms} – F_o)/(F_m – F_o)$	Relative variable fluorescence at the J-step (2 ms)
$V_I = (F_{30ms} – F_o)/(F_m – F_o)$	Relative variable fluorescence at the I-step (30 ms)
$M_o = 4\ (F_{300\mu s} – F_o)/(F_m – F_o)$	Approximated initial slope (in ms^{-1}) of the fluorescence transient V = f(t)
$\varphi_{Po} = TR_o/ABS = 1– F_o/F_m = F_v/F_m$	Maximum quantum yield of primary photochemistry at *t* = 0
$\varphi_{Eo} = ET_o/ABS = F_v/F_m * (1 – V_J)$	Quantum yield for electron transport at t = 0
$F_v/F_o = (F_m – F_o)/F_o$	Maximum primary yield of photochemistry of photosystem II (PSII)
$\psi_{Eo} = ET_o/TR_o = 1– V_J$	Probability (at time 0) that a trapped exciton moves an electron into the electron transport chain beyond Q_A^-
$\varphi_{Ro} = RE_o/ABS = \varphi_{Po}*\psi_{Eo}*\delta_{Ro}$	Quantum yield for the reduction of end acceptors of PSI per photon absorbed
$\rho_{Ro} = RE_o/TR_o = \psi_{Eo}*\delta_{Ro}$	Efficiency with which a trapped exciton can move an electron into the electron transport chain from Q_A to the PSI end electron acceptors
$TR_o/RC = M_o/V_J$	Trapped energy flux per RC at t = 0
$DI_o/RC = ABS/RC – TR_o/RC$	Dissipated energy flux per RC at t = 0
$PI_{abs,total} = (RC/ABS)*(\varphi_{Po}/(1–\varphi_{Po}))$ $*(\psi_{Eo}/(1–\psi_{Eo}))\ *(\delta_{Ro}/(1–\delta_{Ro}))$	Total performance index, measuring the performance up to the PSI end electron acceptors
$MAIP = (F_m – F_o)/(F_I – F_o) – 1$	Maximum amplitude of IP phase
